# Cross-protection and cross-neutralization capacity of ancestral and VOC-matched SARS-CoV-2 adenoviral vector-based vaccines

**DOI:** 10.1038/s41541-023-00737-4

**Published:** 2023-10-04

**Authors:** Sabrina E. Vinzón, María V. Lopez, Eduardo G. A. Cafferata, Ariadna S. Soto, Paula M. Berguer, Luciana Vazquez, Leonora Nusblat, Andrea V. Pontoriero, Eduardo M. Belotti, Natalia R. Salvetti, Diego L. Viale, Ariel E. Vilardo, Martin M. Avaro, Estefanía Benedetti, Mara L. Russo, María E. Dattero, Mauricio Carobene, Maximiliano Sánchez-Lamas, Jimena Afonso, Mauro Heitrich, Alejandro E. Cristófalo, Lisandro H. Otero, Elsa G. Baumeister, Hugo H. Ortega, Alexis Edelstein, Osvaldo L. Podhajcer

**Affiliations:** 1 Laboratorio de Terapia Molecular y Celular, Fundación Instituto Leloir-CONICET; Ciudad Autónoma de Buenos Aires, C1405BWE Buenos Aires, Argentina; 2 Laboratorio de Microbiología e Inmunología Molecular, Fundación Instituto Leloir-CONICET; Ciudad Autónoma de Buenos Aires, C1405BWE Buenos Aires, Argentina; 3 Unidad Operativa Centro de Contención Biológica, ANLIS Dr. Carlos G. Malbrán; Ciudad Autónoma de Buenos Aires, C1282AFF Buenos Aires, Argentina; 4 Servicio Virosis Respiratorias, Laboratorio Nacional de Referencia de Enfermedades Respiratorias Virales, Laboratorio Nacional de Referencia de SARS-CoV-2/COVID-19 OPS/OMS, INEI-ANLIS Dr Carlos G Malbrán; Ciudad Autónoma de Buenos Aires, C1282AFF Buenos Aires, Argentina; 5 Centro de Medicina Comparada, ICiVet-Litoral, Universidad Nacional del Litoral-CONICET; Esperanza, Santa Fe, 3080 Argentina; 6 Instituto de Investigaciones Biomédicas en Retrovirus y SIDA (UBA-CONICET), Ciudad Autónoma de Buenos Aires, C1121ABG Buenos Aires, Argentina; 7 Securitas Biosciences, Montevideo, 11100 Uruguay; 8 Area de Bioterio, Fundación Instituto Leloir; Ciudad Autónoma de Buenos Aires, C1405BWE Buenos Aires, Argentina; 9 Centro de Re-diseño e Ingeniería de Proteínas (CRIP), Universidad Nacional de San Martín, San Martin, Buenos Aires 1650 Argentina; 10 Departamento de Biología Molecular, Facultad de Ciencias Exactas, Físico-Químicas y Naturales, Instituto de Biotecnología Ambiental y Salud, CONICET, Universidad Nacional de Río Cuarto, Córdoba, X5804BYA Argentina

**Keywords:** Viral infection, Vaccines

## Abstract

COVID-19 vaccines were originally designed based on the ancestral Spike protein, but immune escape of emergent Variants of Concern (VOC) jeopardized their efficacy, warranting variant-proof vaccines. Here, we used preclinical rodent models to establish the cross-protective and cross-neutralizing capacity of adenoviral-vectored vaccines expressing VOC-matched Spike. CoroVaxG.3-D.FR, matched to Delta Plus Spike, displayed the highest levels of nAb to the matched VOC and mismatched variants. Cross-protection against viral infection in aged K18-hACE2 mice showed dramatic differences among the different vaccines. While Delta-targeted vaccines fully protected mice from a challenge with Gamma, a Gamma-based vaccine offered only partial protection to Delta challenge. Administration of CorovaxG.3-D.FR in a prime/boost regimen showed that a booster was able to increase the neutralizing capacity of the sera against all variants and fully protect aged K18-hACE2 mice against Omicron BA.1, as a BA.1-targeted vaccine did. The neutralizing capacity of the sera diminished in all cases against Omicron BA.2 and BA.5. Altogether, the data demonstrate that a booster with a vaccine based on an antigenically distant variant, such as Delta or BA.1, has the potential to protect from a wider range of SARS-CoV-2 lineages, although careful surveillance of breakthrough infections will help to evaluate combination vaccines targeting antigenically divergent variants yet to emerge.

## Introduction

It is clear that massive vaccination was the main safeguard to avoid a catastrophic development of the COVID-19 pandemic^1^. The accumulating data on the approved SARS-CoV-2 vaccines indicate that the incidence of severe and critical disease (and deaths) in vaccinated individuals was dramatically reduced compared to the unvaccinated groups, with a positive impact in reducing the burden on healthcare systems around the world^2^. Since extensive follow-up of vaccinated individuals shows that humoral immunity wanes after 3–6 months^3^, even after administration of a boost, the recommendation of the WHO’s advisory group for high-risk groups (older adults, younger adults with comorbidities and immunocompromised individuals, among others) is to receive a boost every 6 months^4^. A recent analysis of 26 studies concluded that hybrid immunity (immunity achieved through a combination of infection and vaccination) confers better protection than vaccination alone^5^ and the strength of hybrid immunity tends to depend on how closely the breakthrough variant matches the one in the vaccine^6^. Although over 13 billion vaccine doses have been administered thus far, with more than 5.5 billion people with a complete primary series, stark discrepancies in vaccine accessibility and affordability across nations have impeded the attainment of the 70 percent target for low-income countries, with only 1 out of 3 people vaccinated with at least one dose as of Jun 21^st^, 2023^1,7,8^. Consequently, these nations persist in enduring the severe consequences inflicted by the pandemic.

The first major transition from the ancestral Wuhan strain in December 2019 occurred only a few months later with the emergence of the D614G mutation in the stalk region of the spike protein^9^. The D614G variant, B.1, outcompeted the ancestral Wuhan lineage (A/B) in 3 months, clearly establishing the capacity of this virus to infect and propagate in human hosts^9,10^. To date, the WHO has classified five lineages as Variants of Concern (VOC), which include Alpha (B.1.1.7), Beta (B.1.351), Gamma (B.1.1.28.1 or P.1), Delta (B.1.617.2) and, more recently, Omicron BA.1 (B.1.1.529)^11^, BA.2 and its derived sublineage XBB1.5 and BA.5 and its derived sublineage BQ1.1^12^. These VOCs posed new challenges, which are currently being addressed by scientists and pharmaceutical companies all over the world^13^.

One of these challenges is related to the waning immunity of vaccines over time and the immune escape of variants, since most VOCs exhibited reduced recognition by previously raised neutralizing antibodies. Beta was the first VOC with a worrisome immune escape profile, although it never spread widely worldwide. Delta, on the other hand, rapidly outcompeted all previous lineages all over the world, and its increased transmissibility jeopardized vaccine efficacy, characterized by the appearance of breakthrough infections^14^. This aspect became even more pressing with the emergence of the Omicron variants, which combined an increased transmissibility with an extensive immune escape profile^12,15^. Breakthrough infection rates are under intense scrutiny and, in fact, severe disease as a result of breakthrough infections has been observed at relevant rates in individuals vaccinated with two doses, slightly improving in those that received a third dose^16^. Since most vaccines are based on the Wu-1 strain, there is a requirement for more robust vaccines that induce a broad immune response against variants.

Immune escape is the result of mutations mainly in the RBD domain, although relevant mutations were also observed in the NTD and at the region proximal to the furin cleavage site^17^. Phylogenetic analyses of Delta and Omicron indicate that these lineages might not directly derive from previous VOCs (Alpha, Beta or Gamma)^15^. The mutational landscape of Omicron BA.1 (29 amino acid substitutions, 3 deletions and 3 insertions only in Spike) and its sublineages^18^ also suggest a long and complex evolutionary process. Despite this, shared mutations arise among different variants by convergent evolution caused by selective pressure^19^. For example, Beta, Gamma and Omicron share the RBD mutations K417N/T, E484K/A and N501Y; the N501Y mutation is also featured by Alpha^20^ and the K417N is also shared by Delta Plus^21^. Delta also shares other mutations with all or some Omicron sublineages, such as T19R/I, G142D, T478K, L452R/Q and P681R/H. These mutations do not only alter Spike antigenicity because of loss of recognition by antibodies elicited by other variants or vaccines, but also modify the immunodominance of the different epitopes in terms of inducing a humoral immune response^22–24^. Moreover, when an individual is repeatedly exposed to SARS-CoV-2 Spike, either by infection or vaccination, the order in which the immune systems encounters the different variants also influence which epitopes are preferentially targeted by B cells and, thus, impact on the cross-protection profile of the sera^25,26^. These effects on immunodominance hierarchy and antigenic imprinting have implications for vaccine strain selection. As with other RNA viruses, such as influenza and HIV, further antigenic drift is expected to happen for SARS-CoV-2^27^, giving rise to novel VOCs and highlighting the need for more broadly effective vaccines.

In a previous study we have shown that an anti-COVID-19 vaccine based on a replication-deficient adenoviral platform named CoroVax induced long-lasting humoral and cellular immune responses^28^. In the present study we produced novel vaccines matched to different VOCs and closely evaluated their capacity to induce an immune response able to protect against mismatched VOCs. Our adenoviral vector-based vaccines were engineered to express VOC-specific, membrane-bound Spike stabilized in alternative prefusion conformations. The vaccines were retargeted to specifically transduce human muscle and dendritic cells. Using these vaccine candidates, we evaluated the neutralization of mice sera to different VOCs, including several Omicron sublineages. We also assessed vaccines’ efficacy to protect mice from a challenge with different VOCs and their capacity in restricting VOCs replication and injury in the lungs, brain, and upper airways.

## Results

### A single dose of CoroVax vaccine variants expressing ancestral or VOC Spikes elicit similar levels of humoral and cellular immunity

**Fig. 1:**
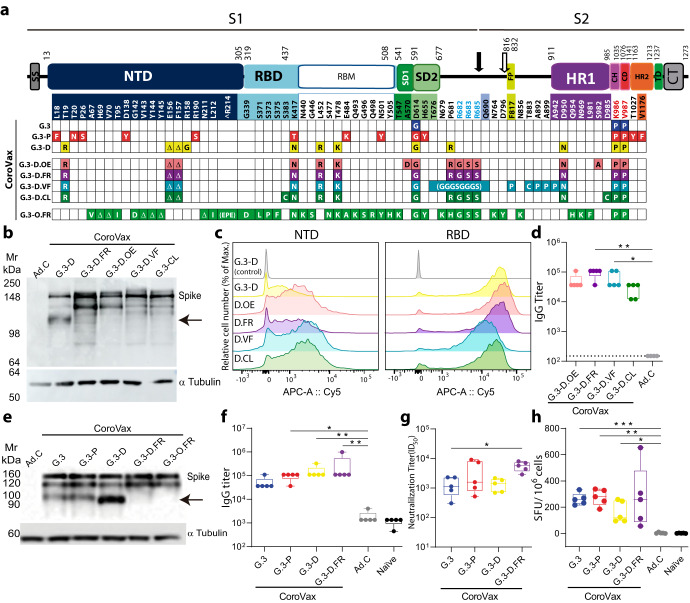
Immunogenicity of the different CoroVax vaccine variants. **a** Schematic representation of the Spike variant expressed by each vaccine. S1 and S2 correspond to the Spike subunits. SS: Signal Sequence, NTD: N-Terminal Domain, RBD: Receptor Binding Domain, RBM: Receptor Binding Motif, SD1: Subdomain 1, SD2: Subdomain 2, FP: Fusion Peptide, HR1: Heptad Repeat 1, CH: Central Helix, CD: Connector Domain, HR2: Heptad Repeat 2, TD: Transmembrane Domain, CT: Cytoplasmic Tail. Solid black arrow: S1/S2 furin cleavage site; Clear arrow: S2’ cleavage site. Mutations in the scheme refer to the A.1 variant. Red letters denote aminoacid changes to stabilize Spike in the prefusion conformation. Blue letters denote aminoacid changes that mutate the furin cleavage motif. **b** Spike expression following in vitro transduction of Hs 729 T cells with the conformation-based vaccines. The arrow shows the absence of the band in Spike constructs expressing a mutated furin cleavage motif. Ad.C corresponds to control Adenovirus. **c** Surface expression of Spike protein in Hs 729 T cells transduced with conformation-based vaccines or vaccine candidates and analyzed by monoclonal antibodies directed to NTD (E7M5X) or RBD domains (40592-R0004). **d** BALB/c mice (*n* = 5) were vaccinated with a single 10^8^ vp dose of each CoroVaxG.3-D conformation-based constructs: Open Enriched (OE), Furin Resistant (FR), 5 (V) proline, Flexibly-Linked, Inter-Protomer bonded (VF) and Closed Locked (CL); sera were collected at 4 weeks postimmunization and assessed for SARS-CoV-2 B.1.617.2 Spike-specific IgG titer. **e** Spike expression of Hs 729 T cells transduced with the candidate vaccines. The arrow shows the furin cleavage motif and the absence of it in furin-resistant Spikes. (**f, g, h**) BALB/c mice (*n* = 5) were vaccinated with a single dose of each CoroVax variant; sera were collected at 4 weeks postimmunization and assessed for (**f**) SARS-CoV-2 B.1 Spike-specific IgG titers (**g**) neutralization titer against matched-PsV and (**h**) cellular immune response. SFU: mean of Spot Forming Units. Ad.C: control adenovirus with no transgene. Differences between experimental groups of animals in **f** and **g** were analyzed by Kruskal–Wallis test followed by Dunn’s multiple comparisons; statistical differences in **h** were analyzed using Brown-Forsythe and Welch ANOVA test with Dunnett T3 *a posteriori*; *p < 0.05, **p < 0.01, ***p < 0.001. The box plots show the median, 25th and 75th percentiles, and the whiskers show the range.

In order to produce the vaccines against the different VOCs, we engineered our previously described vaccine platform, CoroVax^28^, which is based on a hybrid human adenovirus 5, pseudotyped with the fiber knob of adenovirus 3 (hAdV5/3), to code spikes from different SARS-CoV-2 variants, namely B.1 (Wuhan-1 wild type with the D614G mutation), P.1 (Gamma) and B.1.617.2.1 / AY.1 (Delta plus) with the K986P/V987P mutation (PP) to stabilize them in their prefusion conformation (Fig. 1a). The Gamma and Delta plus VOCs were selected based on their high circulation in our region at the onset of the project. In parallel, we wanted to explore the impact of engineering Spike with different mutations that affect the 3D conformation on its immunogenicity, in order to find the best possible immunogen. Thus, we designed 4 additional conformation-based constructs built upon the Delta plus VOC-Spike that should favor, gradually, from more open conformations (with 2 or 3 RBDs in an “up” state) to totally closed conformations (with their 3 RBDs “down”). All constructs incorporated the 2 prolines to stabilize Spike in its prefusion conformation and contained mutations to inactivate the furin cleavage motif: R^682^RAR^68^5 to GSAS mutation for all constructs except for VFLIP, which is furin-resistant by the replacement of the loop containing the furin cleavage site (residues 676–690) with a shorter flexible (Gly-Gly-Gly-Ser) linker^29^ (Fig. 1a). The conformation-based vaccine variants were: (i) CoroVaxG.3-D.OE (Open Enriched), which contains the mutations A570D and S982A, originally found in B.1.1.7 (Alpha) which yield a predominantly open Spike;^30^ (ii) CoroVaxG.3-D.FR (Furin Resistant), which only differs from CoroVaxG.3-D by the RRAR to GSAS mutation, (iii) CoroVaxG.3-D.VF, based on the VFLIP mutations described by Olmedillas et al.^29,31^, which result in a closed yet flexible conformation; and (iv) CoroVaxG.3-D.CL (Closed Locked), which includes mutations S383C and D985C to covalently link the S1 and S2 domains through a disulfide bridge, therefore “closing” the RBDs^32^ (Fig. 1a). Each one of the different vaccine variants built on Delta plus Spike were able to induce the expression of the respective Spike in Hs 729 T human myosarcoma cells (Fig. 1b). As expected, CoroVaxG.3-D.OE/FR/VF/CL that lack an active furin cleavage motif showed the absence of the lowest band (Fig. 1b). Moreover, flow cytometric analysis employing monoclonal antibodies specifically targeting the receptor-binding domain (RBD) and the N-terminal domain (NTD) validated the adequate presentation of distinct Spike proteins on the cellular surface while preserving key conformational epitopes (Fig. 1c and Supplementary Fig. 1a and b); this finding, coupled with the observation that all the constructs elicited a similar degree of immunogenicity, provide evidence for the proper folding and successful exportation to the membrane of the Spike variants. The 4 conformational-based vaccines were compared in a preliminary study for their capacity to induce an anti-Delta Spike immune response in BALB/c mice after i.m. administration of 10^8^ vp. This lower vaccine dose was chosen to allow for the detection of even subtle differences among the conformational-based constructs. The data indicate that, although all conformational-based vaccines induced similar anti-Spike IgG levels (Fig. 1d), CoroVaxG.3-D-FR induced the highest titer (GMTs: CoroVaxG.3-D-OE = 45407; CoroVaxG.3-D-FR = 87780; CoroVaxG.3-D-VF = 56565; CoroVaxG.3-D-CL = 23488; Ad.C = 150) and was therefore selected for further studies.

Next, BALB/c mice were vaccinated with a single dose of 10^9^ vp of the different CoroVax vaccine variants stabilized in their prefusion conformation, i.e. CoroVaxG.3, CoroVaxG.3-P, CoroVaxG.3-D and the selected CoroVaxG.3-D.FR that contains in addition the mutated furin-cleavage motif. All the vaccines were able to induce the expression of their respective Spike in Hs 729 T cells (Fig. 1e). Animal sera exhibited similar IgG binding to the full-length A.1-(Wu-1) Spike among vaccinated groups, pointing to a similar immunogenicity regardless of the Spike variant expressed by the different vaccines (Fig. 1f). When tested against a pseudovirus (PsV) matched to the Spike antigen used in the vaccine, all sera showed a strong neutralizing antibody response (Fig. 1g). However, there was a trend towards CoroVaxG.3-D.FR-vaccinated animals showing a higher neutralizing titer against the homologous PsV (Delta) as compared to the other vaccine candidates. This difference was subtle and only statistically significant for CorovaxG.3-D.FR versus CoroVaxG.3 (Fig. 1g). Although there was some variability in the data, the different vaccines were able to induce a strong cell-mediated anti-SARS-CoV-2 immune response, as measured by interferon-γ ELISpot responses against SARS-CoV-2 spike peptides (Fig. 1h).

### All the vaccines equally cross-protect K18-hACE2 transgenic mice against a challenge with the Gamma VOC

**Fig. 2:**
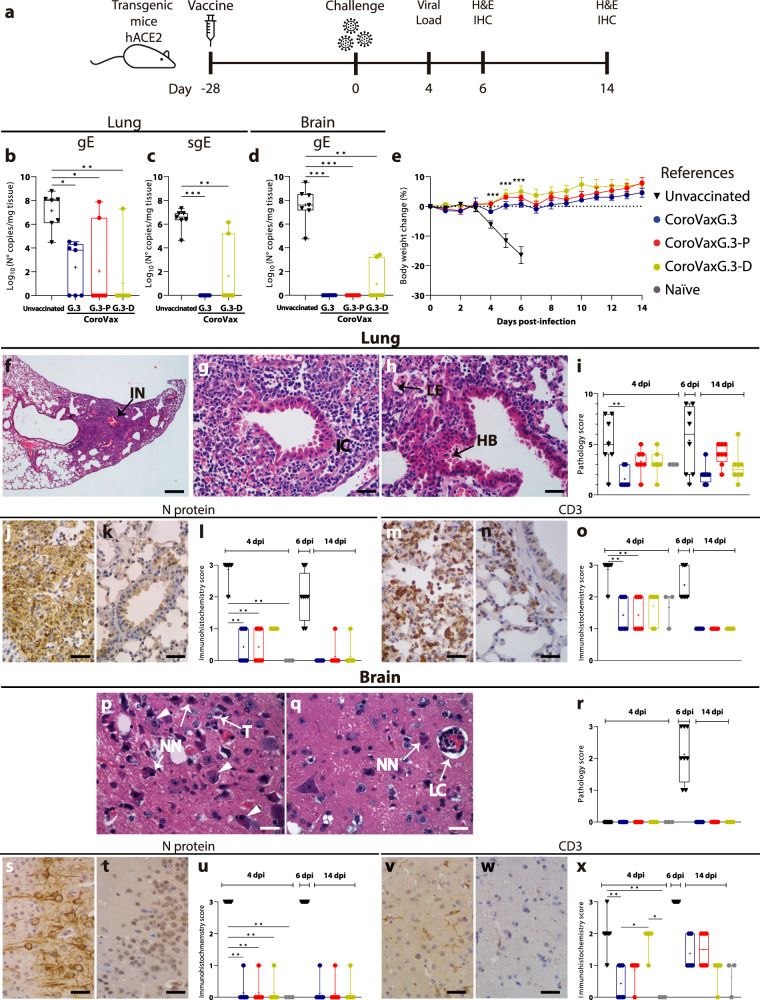
Vaccines’ protection from a challenge with the Gamma VOC. Transgenic mice vaccinated at day −28 were challenged with Gamma at day 0. At day +4 postchallenge, 7 mice of each group were euthanized and all the organs were removed (**a**). Samples of lungs and brains were used to assess viral load by quantitative PCR of gE (**b** and **d**) and sgE (**c**). The rest of the mice were followed to assess weight changes after the challenge (**e**). Immunopathological analysis of the lungs of unvaccinated mice show (**f**) interstitial pneumonia (IN), (**g**) inflammatory cells (IC), and (**h**) loss of epithelial cells (LE) and hyperplastic bronchiole (HB**)** that were used to obtain the pathology score (**i**). Staining of N protein in lungs of unvaccinated (**j** and **l**) and vaccinated mice (**k** and **l**) and CD + 3 infiltrate in unvaccinated (**m** and **o**) and vaccinated mice (**n** and **o**) is also shown. Similar studies on unvaccinated mice brains at day 6 show intraneural inclusion bodies (arrow heads), neuronal necrosis (NN), tigrolysis (T) and lymphoplasmacytic cuffing (LC) (**p** and **q**); the pathology score in the brain is also shown (**r**); staining of N protein in unvaccinated (**s** and **u**) and vaccinated mice (**t** and **u**) and CD3+ cells staining in unvaccinated (**v** and **x**) and vaccinated mice (**w** and **x**) is also shown. Number of mice analyzed: unvaccinated mice at day 4 (*n* = 7) and at day 6 (*n* = 8); all the groups of vaccinated mice: at day 4 (*n* = 7) and at day 14 (*n* = 8); *naïve* mice ranged between 3-4 mice for viral load and histological studies. Image scale bars represents approximately 250 μm for low magnification and 25 μm for 400X images. Differences between experimental groups in **b,** **c** and **d** were analyzed by Kruskal–Wallis test followed by Dunn’s multiple comparisons. Two-way ANOVA with Dunnett *a posteriori* was performed for **e**. Data is presented as mean standard error of the mean (SEM). *p < 0.05, **p < 0.01, ***p < 0.001 vs unvaccinated mice. The box plots in **b, c, d, i, l, o, r, u** and **x** show the median, 25th and 75th percentiles, and the whiskers show the range.

Since the aim of the study was to establish the cross-protective capacity of the different VOC-matched vaccine variants, we next aimed to establish vaccines’ capacity to protect mice from challenges with VOCs that were prevalent at different times in South America. Hence, 6 to 8 months old, K18-hACE2 C57BL/6 J mice, were immunized by administering 10^9^ viral particles of each of the CoroVax-based vaccines and challenged 28 days later intranasally, with a different VOC at the selected dose (Fig. 2a). A group of mice were sacrificed at day 4 to assess viral load in lungs and brains as a readout of local infection and virus dissemination. Histopathological and immunohistochemical analyses were performed in all organs with particular emphasis on the lungs and brain (Fig. 2a).

We initially evaluated vaccine protection from a challenge with the Gamma VOC. As an initial readout of vaccine protection capacity, we evaluated viral load in lungs and brain at day 4 post-challenge (Fig. 2a). All unvaccinated mice showed high viral burden as reflected by the levels of the genomic envelope (gE) gene both in lungs (Fig. 2b) and brain (Fig. 2d). Since gRNA levels might not be a suitable indicator of viral copies in lungs due to a residual contamination by the inoculum, we also assessed subgenomic (sg) RNA that can provide a better correlate of productive viral replication^33^. We observed that sgE levels were also high in the lungs of unvaccinated challenged mice (Fig. 2c). In general, the tested vaccines, i.e. CoroVaxG.3, CoroVaxG.3-P and CoroVaxG.3-D, protected against Gamma infection and replication in affected organs, as evidenced by the significantly reduced viral genome copy number (Fig. 2b, c and d). Interestingly, protection was much larger for the three vaccines in the brain indicating that they were able to prevent virus dissemination, with only two animals vaccinated with CoroVaxG.3-D showing detectable virus in the brain (Fig. 2d). The high viral burden in the lungs and brains was coincidental with body weight loss in the unvaccinated mice; by day 6 all unvaccinated mice had to be euthanized (Fig. 2e). Despite the slight variations we observed in viral load, the three vaccines conferred a similar level of protection and none of the vaccinated mice lost weight throughout the study (Fig. 2e).

Histopathological analysis of unvaccinated mice lungs at 4 days after challenge showed moderate focal to multifocal interstitial pneumonia, peribronchiolar and perivascular infiltration with areas of moderate type II pneumocyte hyperplasia and diffuse congestion (Fig. 2f). Bronchioles were surrounded by inflammatory cells, mainly neutrophils and mononuclear cells (Fig. 2g). Also, thickened alveolar walls and reduced alveolar spaces, with loss of epithelial cells and patchy alveolar oedema, was observed (Fig. 2h). The cumulative lung pathology score demonstrated a slight increase in severity from day 4 to day 6 after challenge, when all unvaccinated mice were euthanized (Fig. 2i). The score was quite similar in vaccinated and naïve mice (Fig. 2i). We observed intense SARS-CoV-2 N protein staining in lungs of unvaccinated mice (Fig. 2j and l) while lungs obtained from vaccinated mice exhibited weaker staining of SARS-CoV-2 N protein that strongly diminished by the end of the experiment at day 14 (Fig. 2k and l). Unvaccinated mice showed numerous infiltrating CD3+ cells at days 4 and 6 (Fig. 2m and o) that were strongly reduced in vaccinated mice (Fig. 2n and o). Histopathological analysis of unvaccinated mice brain at 6 days after challenge evidenced severe injury, including meningoencephalitis with lymphoplasmacytic cuffing, neuronal necrosis, tigrolysis, and intraneuronal inclusion bodies that led to a high pathology score (Fig. 2q, r and s); this was accompanied by intense staining of SARS-CoV-2 N protein (Fig. 2t and v) and abundant CD3+ infiltrate (Fig. 2w and y). On the other hand, vaccinated mice exhibited no brain injury (Fig. 2s) and scarce staining of N protein (Fig. 2u and v) and CD3+ cells (Fig. 2x and y) regardless of the administered vaccine.

### A Gamma-matched vaccine was unable to protect K18-hACE2 transgenic mice against challenge with Delta VOC

**Fig. 3:**
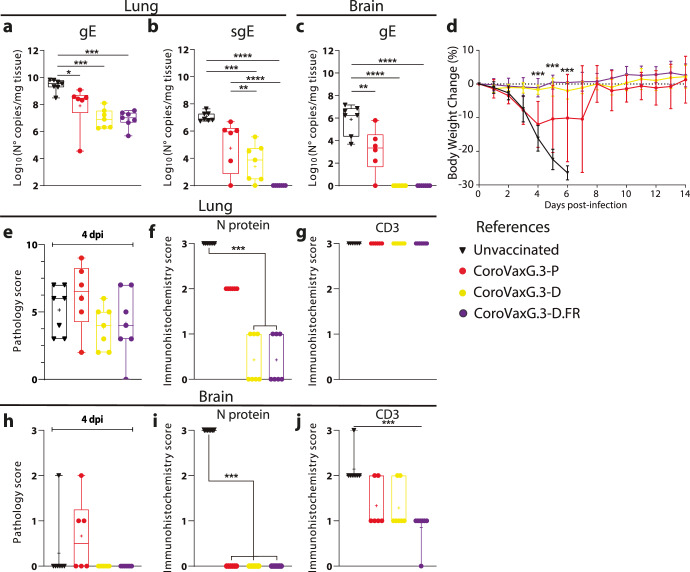
Vaccines’ protection from a challenge with the Delta VOC. Transgenic mice vaccinated at day −28 were challenged with Delta at day 0. At day +4 postchallenge, 7 mice of each group were euthanized and all the organs were removed (see 2a for details). Samples of lungs (**a** and **b**) and brain (**c**) were used to assess viral load by qPCR of gE (**a** and **c**) and sgE (**b**) RNA. The rest of the mice were followed to assess weight changes after the challenge (**d**). Additional samples of all the mice were analyzed to obtain the pathology score in lungs (**e)** and brains (**h**), for expression of **N** protein (**f** and **i**) and CD3 + T cells infiltrate (**g** and **j**). Differences between experimental groups of animals for viral load were analyzed by ANOVA with Bonferroni *a posteriori*. Two-way ANOVA with Dunnett *a posteriori* was performed for analyzing changes in body weight. Data is presented as mean standard error of the mean (SEM). *p* < 0.05, **p < 0.01 and ***p < 0.001. The box plots in **a–c** and **e–j** show the median, 25th and 75th percentiles, and the whiskers show the range.

In order to further analyze the capacity of the different VOC-matched vaccines to cross-protect from different SARS-CoV-2 VOCs, we performed a similar study using the Delta variant to challenge vaccinated mice (see the scheme depicted in Fig. 2a). Since the ancestral strain B.1 was no longer prevalent in our region (and globally) we vaccinated mice with CoroVaxG.3-P and CoroVaxG.3-D and incorporated the modified vaccine version, CoroVaxG.3-D.FR. In samples obtained by day 4 after the challenge, we observed high viral burden in the lungs and brains of control unvaccinated mice with clear differences in mice protection with the different vaccines (Fig. 3a to c). Indeed, viral load assessed using genomic RNA levels (gRNA) of the E gene in lungs was around 1 log lower in mice vaccinated with Delta-matched vaccines compared with the levels in mice vaccinated with the Gamma-matched vaccine (Fig. 3a). Assessment of sgRNA E levels demonstrated that none of the mice vaccinated with CoroVaxG.3-D.FR showed viral replication in lungs (Fig. 3b). In addition, none of the mice vaccinated with the Delta-matched vaccines exhibited viral replication in brain, whereas some replication could be still observed in brains of mice vaccinated with the Gamma-matched CoroVaxG.3-P vaccine (Fig. 3c). Thus, the Gamma-matched vaccine was unable to fully protect mice from infection with Delta.

The high viral burden observed in lungs and brains was coincidental with a dramatic weight loss that started at day 3 leading to the euthanasia of all unvaccinated mice by day 6 (Fig. 3d). Noteworthy, several mice vaccinated with the Gamma-matched vaccine showed weight loss between days 3 to 7 (Fig. 3d). Although some of the mice recovered, 2 out of 7 mice continued to lose weight leading to their euthanasia by day 6 (Fig. 3d). Contrary to that, all mice vaccinated with the two Delta-matched vaccines showed no weight loss and remained in good health until the end of the study (Fig. 3d). Consistent with the partial protection from challenge of the CoroVaxG.3-P vaccine, analysis of the neutralizing antibody titer in sera collected prior to challenge showed that mice vaccinated with CorovaxG.3-D.FR elicited significantly higher levels of anti-SARS-CoV-2 Delta nAbs than animals inoculated with the CorovaxG.3-P candidate (Supplementary Fig. 2).

Histopathological analysis of samples obtained by day 4 showed that lungs of unvaccinated K18-hACE2 mice challenged with the Delta VOC exhibited tissue injury features and pathology scores similar to those observed in unvaccinated mice challenged with Gamma (Fig. 3e). Interestingly, the pathological score did not differ between unvaccinated mice and mice vaccinated with the Gamma-matched vaccine (Fig. 3e); in coincidence with the full protection from challenge with the Delta VOC, mice vaccinated with the two Delta-matched vaccines showed a slightly lower lung pathology score compared to the control and the Gamma-matched vaccine (Fig. 3e). In close coincidence with the previous data, we observed an intense staining of the SARS-CoV-2 N protein in unvaccinated mice and in mice vaccinated with the Gamma-matched vaccine, which differed with the statistically significant lower levels of N staining in mice vaccinated with the Delta-matched vaccines (Fig. 3f). Of note, we observed no difference in CD3+ infiltration among the different groups (Fig. 3g). Brain injury was observed in some unvaccinated mice and in mice vaccinated with the Gamma-matched vaccine (Fig. 3h). Intense staining of the N protein was observed only in unvaccinated mice (Fig. 3i), while no major difference was observed in CD3+ infiltration with the exception of the CoroVaxG.3-D.FR vaccinated group that showed a statistically significant decrease in CD3+ infiltrating lymphocytes (Fig. 3j). Histological analysis of unchallenged mice vaccinated with any of the vaccines showed the complete absence of tissue injury either in lungs or brain (Supplementary Fig. 3). The whole data indicate that the Gamma Spike-based vaccine was unable to fully cross-protect mice from a challenge with the Delta VOC.

### Comparative studies with an Omicron-matched vaccine in prime/boost regimens

**Fig. 4:**
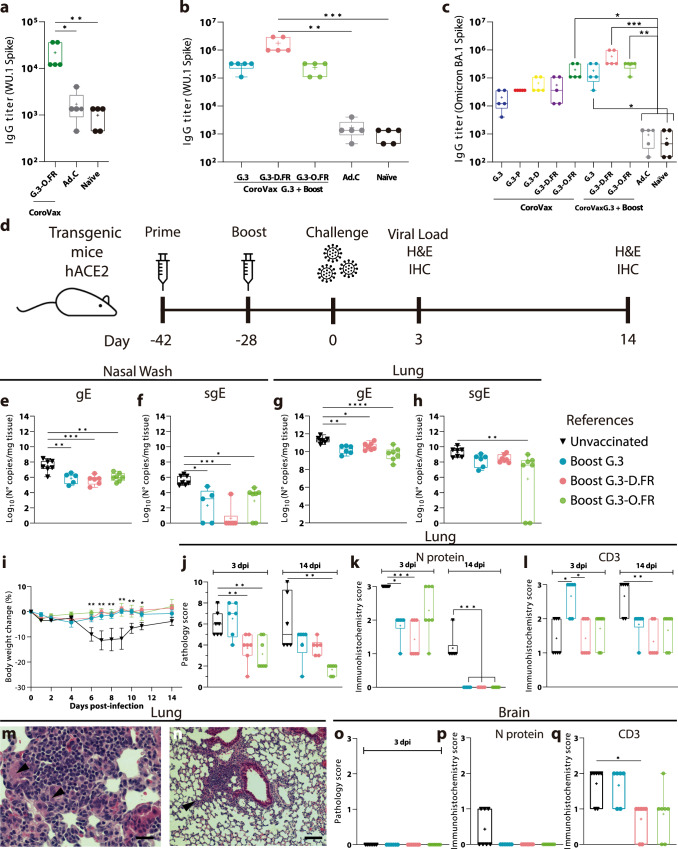
Vaccines’ protection from a challenge with the Omicron BA.1 VOC. BALB/c mice were immunized with CoroVaxG.3-O.FR (**a** and **c**) or with a prime/boost scheme (**b** and **c**). Sera from vaccinated mice was used in an ELISA to assess IgG levels against Wu-1 Spike (**a** and **b**) and Omicron BA.1 Spike (**c**). Transgenic mice vaccinated at day −42 with a priming dose and at day −28 with a booster dose were challenged with Omicron at day 0. At day +3 postchallenge, 6-7 mice of each group were euthanized and all the organs were removed (**d**). Samples of nasal washes (**e** and **f**) and lungs (**g** and **h**) were used to assess viral load by qPCR of gE (**e** and **g**) and sgE (**f** and **h**). The rest of the mice were followed to assess weight changes after the challenge (**i**). Samples of lungs at day 3 after the challenge were analyzed to obtain the pathology score (**j**), for expression of N protein (**k**) and CD3 + T cells (**l**). The presence of syncytial cells in the lungs of unvaccinated (**m**) and vaccinated mice (**n**) is shown (arrowheads). Samples of brains at day 3 postchallenge were analyzed to obtain the pathology score (**o**), for expression of N protein (**p**) and CD3 + T cells (**q**). Number of mice analyzed: unvaccinated mice at day 3 (*n* = 7), while for vaccinated mice we analyzed *n* = 6 for the boost with CoroVaxG.3 and *n* = 7 for the other two groups of boosted mice; at day 14 for all groups (*n* = 6); 3 *naïve* mice were assessed for viral load and histological studies. Differences between experimental groups of animals for gE and sgE were analyzed by ANOVA with Bonferroni *a posteriori* and Kruskal-Wallis test with Dunn´s multiple comparisons *a posteriori*, respectively. Two-way ANOVA with Dunnett *a posteriori* was used for analyzing changes in body weight. Data is presented as mean standard error of mean (SEM). *p* < 0.05, **p < 0.01 and ***p < 0.001. The box plots in **a**–**c, e**–**l** and **o**–**q** show the median, 25th and 75th percentiles, and the whiskers show the range.

During the course of these studies, SARS-CoV-2 Omicron BA.1 emerged in our region and rapidly outcompeted Delta. In this context, we decided to produce an Omicron BA.1-matched vaccine (CoroVaxG.3-O.FR) (see Fig. 1a) to compare with the previous ones, using a prime/boost regimen in close resemblance of a real-world scenario. In the set-up studies, we found that sera obtained after vaccination with a single dose of CoroVaxG.3-O.FR showed less IgG binding to Wu-1 Spike, compared to the rest of the vaccine variants (Fig. 4a; compare with Fig. 1f).

Next, we moved to the prime/boost scheme and administered either CorovaxG.3 that expresses ancestral B.1 Spike, CorovaxG.3-D.FR matched to Delta Plus Spike or CorovaxG.3-O.FR matched to Omicron BA.1 Spike as a booster following a priming dose with CoroVaxG.3. The combination of CoroVaxG.3 followed by CoroVaxG.3-D.FR showed the best immunogenicity using Wu-1 as the target antigen (Fig. 4b). Sera from mice immunized with the same prime/boost regimen were analyzed using Omicron BA.1 Spike as a target. Interestingly, the combination of CoroVaxG.3 followed by CoroVaxG.3-D.FR also exhibited the largest statistically significant IgG levels against Omicron BA.1 Spike even compared to a booster with the matched CoroVaxG.3-O.FR (Fig. 4c). This result was surprising since, as expected, IgG levels against Omicron BA.1 Spike were the highest after a single dose vaccination with the matched Omicron BA.1 vaccine (Fig. 4c).

In order to establish the protective capacity of the different prime/boost combinations, aged transgenic K18-hACE2 mice were boosted either with CorovaxG.3, CorovaxG.3-D.FR or CorovaxG.3-O.FR 28 days after a priming dose with CoroVaxG.3, followed by mice challenge 14 days later with the Omicron BA.1 VOC (Fig. 4d). Using gE and sgE gene levels as a readout of viral replication, we observed high viral burden in nasal washes and lungs of unvaccinated mice at 3 days after challenge (Fig. 4e to h). In general, a slight, but statistically significant decrease in viral burden was observed in nasal washes and lungs in mice vaccinated with any of the prime/boost regimens using either gE or sgE levels as a readout (Fig. 4e to h) with the exception of sgE levels in the lung that were statistically significantly different only in mice vaccinated with the Omicron-matched booster (Fig. 4g). Confirming that compared to the previous VOC, Omicron caused decreased disease severity upon infection in mice^34^, follow up of the unvaccinated mice showed that 4/6 mice lost more than 10% of body weight and we were forced to euthanize only one of them (Fig. 4i). On the other hand, 1/6 mice vaccinated with a booster dose of CoroVaxG.3 lost more than 10% body weight, while none of the mice receiving a booster of CoroVaxG.3-D.FR or CoroVaxG.3-O.FR lost more than 10% weight after the challenge (Fig. 4i).

We observed similar lung injury in unvaccinated mice and in mice boosted with CoroVaxG.3 at 3 days post infection (dpi) (Fig. 4j), while mice boosted with the Delta and the Omicron Spike-matched vaccines were better protected from the challenge (Fig. 4j). At 14 dpi mice boosted with CoroVaxG.3-O.FR were able to reduce lung injury, while CoroVaxG.3 and -D.FR showed a tendency to decreased lung injury (Fig. 4j). N protein levels were the highest in lungs of unvaccinated mice compared to vaccinated mice, although by day 14 after challenge staining diminished in unvaccinated mice and were undetectable in vaccinated mice (Fig. 4k). CD3+ infiltration increased in lungs of unvaccinated mice from day 3 to day 14 (Fig. 4l), while CD3+ infiltrate was high at day 3 in mice vaccinated with CoroVaxG.3 but diminished in all vaccinated mice by day 14 after challenge (Fig. 4l). Although the lung lesions features were similar to those generated by Gamma and Delta challenge, the challenge with Omicron induced numerous syncytia at day 14 after challenge in multifocal interstitial pneumonia areas (Fig. 4m); on the other hand, vaccinated mice showed only few syncytia and limited to perivascular and peribronchiolar areas (Fig. 4n). No brain lesions were observed at day 3 after challenge in any of the groups (Fig. 4o) which was accompanied by low N staining only in unvaccinated mice (Fig. 4p) and lower levels of CD3+ staining in mice boosted with the Delta-matched vaccine compared to unvaccinated mice (Fig. 4q). Thus, a booster with the Delta-matched CoroVaxG.3-D.FR vaccine was as good as a booster with the Omicron-matched CoroVaxG.3-O.FR vaccine, to control Omicron BA.1 replication, dissemination and induction of lung and brain injury.

### Assessment of the cross-neutralization capacity of mice sera after a prime/boost vaccination

**Fig. 5:**
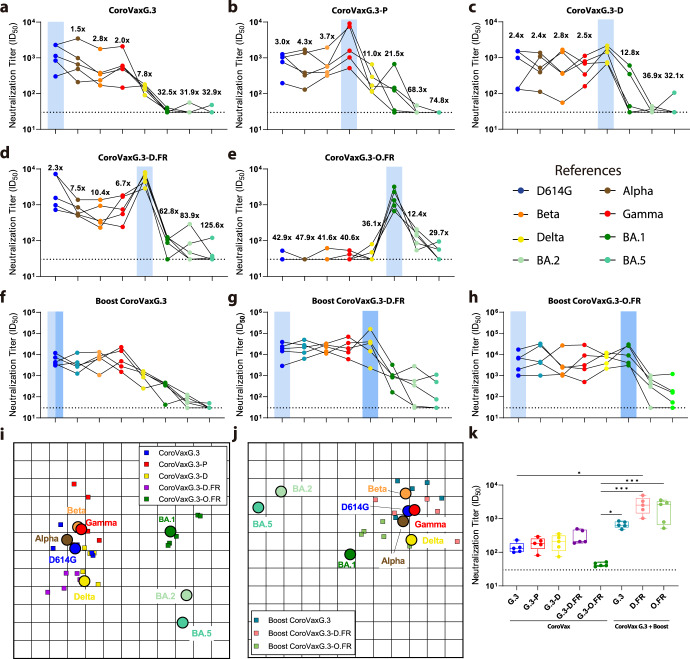
Cross-neutralization of VOC-matched pseudoviruses by sera from CoroVax vaccinated mice. Sera from BALB/c mice were tested against pseudoviruses bearing B.1 (D614G), B.1.1.7 (Alpha), B.1.351 (Beta), P.1 (Gamma), B.1.617.2 (Delta), B.1.1.529.1 (Omicron BA.1), B.1.1.529.2 (Omicron BA.2), and B.1.1.529.5 (Omicron BA.5) spikes, and the calculated ID_50_s are depicted in the graph. The dashed line indicates the limit of detection. The differences in neutralization of different variant viruses are indicated by horizontal lines, and the fold differences in neutralization GMTs are shown. Mice were immunized with a single injection of (**a**) CorovaxG.3, (**b**) CorovaxG.3-P, (**c**) CorovaxG.3-D, (**d**) CorovaxG.3-D.FR, (**e**) CorovaxG.3-O.FR. For prime/boost experiments mice were immunized with a dose of CorovaxG.3 followed by a boost 28 d later of either (**f**) CoroVaxG.3, (**g**) CoroVaxG.3-D.FR or (**h**) CoroVaxG.3-O.FR. **i** Antigenic map of SARS-CoV-2 VOCs based on sera of mice vaccinated with a single dose of CoroVax candidates. SARS-CoV-2 variants are displayed as circles and sera are represented by squares. Each square corresponds to serum of one animal. Each grid square represents 1 antigenic unit and equals a 2-fold change in neutralization titer. (**j**) Antigenic map of SARS-CoV-2 VOCs based on sera of mice vaccinated with a prime/boost regimen of CoroVax candidates. The circles more closely together evidence higher cross-neutralization. ID50s used to build these maps were extracted from Supplementary Table 1. **k** Cross-neutralization titer was calculated as the GMT against all VOCs except the matched strain. The box plots shows the median, 25th and 75th percentiles, and the whiskers show the range.

In order to further evaluate how broad is the protection profile, we evaluated the cross-neutralization capacity of each vaccine as a single dose or in the prime-boost setting described above using PsVs pseudotyped with Spikes corresponding to the different VOCs (Supplementary Fig. 4). The initial analysis of BALB/c mice vaccinated with a single dose showed that, when considering all VOCs arisen before Omicron (Alpha to Delta), all vaccine candidates elicited a significant nAb response against all of the VOCs, either homologous or heterologous, although with subtle differences (Fig. 5a–d, Supplementary Table 1). CoroVaxG.3-D.FR displayed the highest levels of neutralization not only to its matched PsV but also to the ancestral B.1 PsV, performing even better than its matched vaccine, CoroVaxG.3 (Figs. 5a, d and Supplementary Table 1). CoroVaxG.3 exhibited no detectable neutralizing activity against Omicron BA.1 (Fig. 5a and Supplementary Table 1); in comparison, sera from mice vaccinated with a single dose of Gamma- and Delta-Spike-based vaccines induced a slightly better ID_50_ GMT against Omicron BA.1 (Fig. 5b–d and Supplementary Table 1). Conversely, a single dose of CorovaxG.3-O.FR failed to elicit nAbs against any of the VOCs with the exception of its matched PsV (Fig. 5e and Supplementary Table 1). Interestingly, when tested against emerging Omicron VOCs, such as BA.2 and BA.5 which are the parental lineages of XBB.1.5 and BQ.1.1, respectively, all the single-dose vaccinations were unsuccessful in cross-neutralizing the corresponding pseudoviruses and even CoroVaxG3-O.FR, based on an Omicron variant, did not perform any better against BA.2 or BA.5 than CoroVaxG3-D.FR (Fig. 5d, e and Supplementary Table 1).

Although boosting with either CorovaxG.3, CorovaxG.3-D.FR or CorovaxG.3-O.FR increased the ID_50_ GMTs against all the tested VOCs, the detailed cross-neutralization profile of the different boosters showed significant differences (Fig. 5f–h and Supplementary Table 1). A homologous boost with CorovaxG.3 raised neutralization titers to high levels for some VOCs (GMT against D614G, Alpha, Beta and Gamma in the range of 3504–5844) but only to moderately high against Delta (GMT of 898) and showed relatively low titers against all tested Omicron lineages (GMT in the range of 33–262) (Supplementary Table 1). A heterologous boost with CorovaxG.3-D.FR showed a much more advantageous profile, with extremely high titers against most VOCs (GMT against D614G, Alpha, Beta, Gamma and Delta in the range of 14869–23333) and relatively high titers against Omicron BA.1 (GMT of 852) (Supplementary Table 1). On the other hand, a heterologous boost with CorovaxG.3-O.FR displayed a significantly higher neutralization against BA.1 (GMT of 9662) and also showed a high cross-neutralization to other VOCs (GMT against D614G, Alpha, Beta, Gamma and Delta in the range of 2876–6903), but was generally not better than the homologous CoroVaxG.3 in boosting the response against these variants (Supplementary Table 1). On the contrary, neither CorovaxG.3-D.FR nor CorovaxG.3-O.FR could boost the titer of nAb to BA.2 or BA.5 to high levels, and both boosters showed comparable cross-neutralization profiles to these Omicron variants (Fig. 5f–h and Supplementary Table 1).

In order to better dissect the cross-neutralization potential of the different vaccines and how they relate to the different VOCs, we used the Pseudovirus-Based Neutralization Assay (PBNA) data to construct SARS-CoV-2 antigenic maps (Fig. 5I, j). As expected, sera of single dose-vaccinated mice tend to cluster in the proximity of the variant they were raised against (Fig. 5i). The ancestral and Alpha spikes cluster together, as do the Beta and Gamma VOCs, and Delta stands somehow separate from those microclusters; still, the five variants are located within 2 antigenic units (AU) of each other (1 AU = 2-fold change in neutralization titer), pointing to a high degree of antigenic similarity. On the other hand, Omicron BA.1 is at least 4 AU away from the antigens in the “original” SARS-CoV-2 cluster, indicating a > 16-fold change in neutralization titer, confirming that Omicron BA.1 would be deemed as antigenically distant from the other VOCs. Interestingly, although BA.2 and BA.5 are also deemed as Omicron variants, they are roughly as far away from BA.1 than from the “original” SARS-CoV-2 cluster, with a distance of approximately 4 AU from BA.1 and 5 AU from Delta (Fig. 5i). As expected, the antigenic map resulting from the analysis of the prime/boost sera (Fig. 5j) reduced the distance among VOCs, pointing to the fact that these sera have a more balanced cross-neutralization profile than single-dose vaccination sera. However, all boosted sera are still located far from BA.2 and BA.5 variants, reflecting their lack of cross-neutralization to these more recently emerged variants.

To shed some light on the overall improvement in the cross-neutralization capacity of sera obtained from the different prime/boost schemes, we determined the capacity of each vaccine to induce specifically cross-neutralizing antibodies. As expected, sera obtained after boosting showed a broader cross-neutralizing response than single-dose sera (Fig. 5k). Although not statistically significant, it was of note that boosting with an antigenically different vaccine, such as CorovaxG.3-D.FR or CorovaxG.3-O.FR after priming mice with CoroVaxG.3, induced a broader cross-neutralizing response than using the same vaccine (CoroVaxG.3) in the prime and boost setting.

## Discussion

The emergence of SARS-CoV-2 strains evading immunity from vaccination or prior infection, especially the Delta and Omicron variants of concern, jeopardized the efficacy of vaccines designed against the ancestral SARS-CoV-2 and posed an urgent need of updated variant-proof vaccines^35^. With the aim to select a candidate for broad coverage of SARS-CoV-2 variants, we designed adenoviral-based vaccines expressing ancestral or VOCs-matched prefusion-stabilized, membrane-bound Spike, expressing an active or mutated furin-cleavage motif. The different vaccine variants were intensively assessed in preclinical models for their cross-neutralizing capacity against a large panel of PsV expressing matched and mismatched Spike and their cross-protection against challenge with authentic VOCs.

All variant-targeted vaccines induced a comparable response in terms of humoral immunity and neutralization of their matched strain. Of note, a higher IgG titer and homologous ID_50_ was measured in sera of animals vaccinated with the furin-resistant CoroVaxG.3-D.FR candidate than with its cleavable CoroVaxG.3-D counterpart; this is consistent with other studies showing that ablation of the furin-cleavage motif increases stability and immunogenicity of full-length Spike^36,37^. Interestingly, while each VOC-matched vaccine with an active furin-cleavage motif displayed the best nAb GMT for its own strain (CorovaxG.3 for D614G, CoroVaxG.3-P for Gamma and CoroVaxG.3-D for Delta), the furin resistant CorovaxG3-D.FR outperformed CoroVaxG.3 in D614G neutralization. Therefore, the RRAR to GSAS mutation was also considered for CoroVaxG.3-O.FR design.

We observed that 100% of K18-hACE2 transgenic mice vaccinated with any of the tested vaccines were protected from a challenge with the Gamma VOC, including a vaccine expressing Delta plus Spike. On the contrary, K18-hACE2 mice challenged with the Delta VOC were fully protected when vaccinated with either of the Delta plus-matched formulations but not when vaccinated with CoroVaxG.3-P, with some of the animals showing extreme weight loss and an overall impaired health condition that led to euthanasia. Thus, the Delta plus-matched vaccines showed a better cross-protection against a challenge with a nonmatched VOC. The viral loads and histological studies were consistent with the fact that the Delta-targeted vaccines cross-protected better against a mismatched VOC, such as Gamma, than the opposite. Indeed, only 2/12 mice vaccinated with the Delta Plus Spike-expressing vaccines (regardless of whether they expressed a furin active or mutated motif) showed viral replication in the brain after challenge with Gamma (see Fig. 2d); on the contrary, 5/6 K18-hACE2 mice vaccinated with a Gamma Spike-expressing vaccine were unable to block Delta replication in brain (see Fig. 3c).

In a study performed prior to the emergence of Delta, Amanat et al. showed that mice vaccinated with adjuvanted Spike matched to the ancestral Wuhan strain, Alpha, Beta or Gamma were equally able to inhibit viral replication of the ancestral Wuhan strain in lungs^38^ and a Gamma-based vaccine showed a good cross-neutralization profile. However, when considering cross-neutralization to Delta, other studies showed that Gamma-convalescent sera were particularly deficient in neutralizing Delta, in fact more than any other convalescent or vaccine sera^39,40^, making them more susceptible to breakthrough infection with this VOC. In our study, we also demonstrated that mice vaccinated with a Gamma-matched Spike were susceptible to Delta infection, whereas mice vaccinated with a Delta Plus-matched Spike were completely protected from a challenge with the Gamma strain. Although this lack of reciprocity may look counterintuitive at first, it has been reported that different strains elicit antibody responses with altered immunodominance hierarchy^22,23,41^. In particular, mutations 417 N/T, 484 K and 501Y in RBD, hallmarks of Beta and Gamma, shift the predominant B cell clones towards class 1 and 2 epitopes, compared to the immune response to ancestral SARS-CoV-2^42,43^. Therefore, Beta and Gamma convalescent sera are particularly sensitive to the 417 K mutation, whereas Wu-1 convalescent sera are relatively resistant to 417 N/T mutation^21,22^. However, the Delta-matched vaccines used in this study are in fact based on the AY.1 Delta Plus Spike, which harbors a K417N mutation in RBD on top of the Delta-specific modifications. Thus, it would be expected that CorovaxG.3-D and CorovaxG.3-D.FR elicit nAbs reactive towards Delta-specific epitopes, without such a drastic drop in Gamma/Beta-specific nAbs. Interestingly, the K417N mutation is shared not only by Delta Plus and Beta, but also by Omicron sublineages^21^. A booster with CoroVaxG.3-D.FR on mice primed with CoroVaxG.3 was able to protect aged K18-hACE2 mice from a challenge with Omicron BA.1 similarly to a boost with the Omicron-matched CoroVaxG.3-O.FR vaccine, whereas a boost with the ancestral CoroVaxG.3 could not confer full protection, supporting the idea of a wider protective capacity of CoroVaxG.3-D.FR.

Antigenic cartography of the sera of vaccinated animals showed that mice who received a booster broadened their antibody repertoire. Pre-Omicron VOCs could be grouped into an antigenic cluster, which contains D614G, Alpha, Beta, Gamma and Delta. However, although the different Omicron sub-variants included in this study are distinct to preomicron variants, they did not cluster together, showing that they are also antigenically different from each other. Our study does not include sera from BA.2 or BA.5 targeted vaccines, so positioning of these variants with strong immune escape is restricted by their low cross-reactivity to the included sera, since most of them displayed neutralization titers for these variants below the detection limit. Nevertheless, the relative position and the distance to BA.1 and pre-omicron variants of our single-dose antigenic map is consistent with antigenic maps based on hamster single-exposure sera or human single or multiexposure sera, which also show that BA.2 and BA.5 exhibit unique antigenic characteristics that place them between pre-omicron variants and BA.1^44–47^. A booster with CoroVaxG.3-O.FR showed a balanced neutralizing response, with similar titers against preomicron VOCs and BA.1, whereas a booster with CoroVaxG.3-D.FR displayed the broadest cross-neutralizing activity with extremely high titers against preomicron VOCs and more modest neutralization to BA.1. None of the boosts was effective in cross-neutralizing BA.2 or BA.5 PSVs, consistent with previous reports that BA.2 and BA.4/5 evade antibodies elicited by BA.1^48–50^. Recently, bivalent COVID-19 booster vaccines combining the ancestral strain of SARS-CoV-2 and an Omicron variant were approved in the US and the UK, either based on BA.1 or BA.4/BA.5^51^. Other studies suggested that the Omicron-based boosters offer about the same degree of protection against BA.4 and BA.5 than updated vaccines based on the Beta or Delta variants^52^; moreover, BA.1 breakthrough infection expands cross-reactivity to these more recently emerged VOCs only to a certain extent, not greater than a breakthrough infection with the Delta variant^53^. Recent data suggested that exposure to three antigenically similar or to two antigenically distinct variants induced a great breadth of response, cross-neutralizing nonrelated variants^47^, but this expanded cross-neutralization is not extended to more recent omicron lineages such as XBB.1^44^.

The Delta Plus variant is relatively distant from other variants in the pre-omicron cluster and also shares key mutations with Omicron sublineages, such as K417N or the L452R mutation with BA.4/5 and BQ.1, which is absent in BA.1 and has been heavily associated with immune escape. Thus, it is likely that a Delta Plus matched vaccine is distinct enough with respect to a vaccine based on the ancestral SARS-CoV-2, and therefore can elicit nAbs capable of cross-neutralizing other Omicron subvariants to the same extent than a BA.1-based vaccine can. Noteworthy, we showed that a booster with CoroVaxG.3-D.FR elicited a slightly higher titer of total BA.1-reactive IgG than a booster with CoroVaxG.3-O.FR itself. In this regard, previous studies have shown that infection with Delta (and Beta) was able to induce an improved cross-reactive Fc effector function against global VOCs compared with individuals infected with the ancestral strain or vaccinated with the available ancestral strain-based vaccines^54^. Also, Omicron BA.1 infected individuals sera showed a more restricted cross-reactive Fc effector function suggesting that VOC-related spike sequence plays a role in the elicitation of ADCC^55^.

Up to June 2023, only 34.3% of people in low-income countries have received a single vaccine dose^7^, bringing in a *scenario* where vaccines still have to be designed with a naïve population in mind, as well as already vaccinated individuals. Moreover, follow-up of vaccinated individuals even after boosting, indicates that neutralizing antibodies wane after 3–6 months^3^, leading to current guidelines of the WHO’s advisory group to vaccinate high-risk individuals every 6 months and the general population yearly. Monovalent BA.1 vaccines show very limited cross-reactivity, not only in our study but in others^56,57^, while other studies have shown that a Delta-targeted vaccine or Delta infection can elicit cross-neutralizing antibodies against Delta and Omicron, whereas the reversal is not true^58,59^. Based on our data that a booster with CoroVaxG.3-D.FR induced nAbs against mismatched variants and protects mice from heterologous SARS-CoV-2 infection and dissemination to the brain, we suggest that a booster with CoroVaxG.3-D.FR could provide a long-lasting and broad immunization against some SARS-CoV-2 strains, and would also be a suitable candidate for unvaccinated individuals. However, additional updates of COVID-19 vaccines might be needed, including variants representing currently circulating strains and variants yet to emerge.

## Methods

### Study design

The aim of this study was to design COVID-VOC-matched vaccines in order to search for a potential candidate to enter clinical trials that will confer wider cross-protection and cross-neutralization capacity. Using our targeted adenoviral vector-based platform CoroVaxG.3 that was designed to induce the immunodominance of the transgene, we engineered Spike corresponding to each one of the SARS-CoV-2 VOCs; we also constructed pseudoviruses (PsVs) against each one of the VOCs. For the in vivo studies in animal models, we selected those VOC that were prevalent in our region at the time of the study. As a first approach, we aimed to establish if there is any difference in the capacity of each one of the vaccines to induce a humoral response in BALB/c mice against the ancestral strain Spike. In the next series of studies our aim was to establish whether VOC-matched vaccines would be able to cross-protect from a mismatched VOC. These studies were performed in aged K18-hACE2 transgenic mice in the understanding that older people are more likely to develop serious illness. All the studies aiming to assess cross-protection, viral load (genomic and subgenomic) and histopathology/immunohistochemistry were blinded, including the mean histopathological score that was performed by a veterinary pathologist.

### Ethics and biosafety studies

All the animal studies were approved by the Institutional Animal Care and Use Committee (IACUC) of Fundacion Instituto Leloir under CICUAL protocol ID 97. BALB/c mice were obtained from the Animal Facility of the Faculty of Veterinary of University of La Plata. Transgenic K18/hACE2 mice were purchased from Jackson Laboratories (Bar Harbour, Maine), transported to Argentina and housed and expanded at the enforced BSL2 Facility of the Center for Comparative Medicine of the National University of Litoral. This facility complies with the requirements of the National Service of Agriculture & Food Security and Quality (SENASA), the National Agency for Medicines, Food and Medical Technology (ANMAT) and with the OECD principles of GLP. All the studies that involved the use of SARS-CoV-2 live strains were approved for use at the BSL3A Biological Containment Area for small Animals of Instituto Malbran by the Biosafety Committee, the Scientific Coordination and the IACUC of the same Institute. All the personnel involved in the animal studies with live virus in the BSL3A area wore powered air-purifying respirators, Tyvek suits, and were double gloved. The BSLA3 laboratory has been designed according to the safety requirements recommended by the Biosafety in Microbiological and Biomedical Laboratories (BMBL) Manual, the U.S. Department of Health and Human Services, the Public Health Service, the Centers for Disease Control and Prevention (CDC), and the National Institutes of Health (NIH).

### Reagents and cells

HEK293T (CRL-3216) and Hs 729 T (HTB-153) cell lines were obtained from the ATCC (Manassas, VA, USA). HEK293 cells were purchased from Microbix Biosystems Inc (Mississauga, ON, Canada); 911 cells and HEK293T-hACE2 cells were already described^28^. All the cell lines were grown in the recommended medium supplemented with 10% of FBS (Natocor, Cordoba, Argentina), 2 mM glutamine, 100 U/mL penicillin and 100 μg/mL streptomycin and maintained in a 37 ˚C atmosphere containing 5% CO_2_.

### Vaccines design and production

The sequence of the reference Spike protein corresponding to each VOC was extracted from the Outbreak.info database (https://outbreak.info/situation-reports)^60^. The sequence of the B.1 ancestral strain was already described^28^. To construct each vaccine version, the plasmid pS-Spike(D614G)-PP^28^, was restricted either with a combination of XhoI/EcoRV or XhoI/SwaI, to delete the Spike sequence to be replaced. Simultaneously, the Spike region was amplified in several overlapping fragments using primers containing the amino-acid changes to be introduced. To build each pShuttle-Spike version, the vector fragment and the Spike fragments were reassembled using Gibson assembly^61^; each pShuttle plasmid version was confirmed by BS-sequencing (CELEMICS, Seul, Korea). The non-replicating adenoviruses were constructed and produced as described^28^. Basically, the plasmid pS-Spike was linearized with PmeI and co-transformed with E1 deleted adenoviral backbone vectors in electrocompetent BJ5183 bacteria. The identity of the plasmids was confirmed by sequencing. The recombinant DNAs were linearized with PacI and transfected into 911 cells. The viruses were propagated in HEK293 cells in CellSTACK® cell culture chambers (Corning, Corning, NY, USA), purified by double CsCl density gradient centrifugation and stored in 10% glycerol in single-use aliquots at −80 °C.

### Assessment of Spike expression

To assess Spike expression by western blots, 1 × 10^6^ Hs 729 T cells were seeded and cultured in 6-well plates overnight. The following day cells were transduced with the different adenoviral constructs at MOI 100 for 48 h. At the end, cells were washed twice with ice-cold PBS and lysed in a 2X Laemmli sample buffer. Protein extracts were separated, transferred to nitrocellulose membranes and probed with an anti-spike Ab (40150-T62, Sino Biological Wayne, PA, USA) and anti-α-tubulin Ab (Ab18251; Abcam, Cambridge, UK). Spike expression was detected and quantified by densitometry using the ImageJ software 1.53. The uncropped and unprocessed images used to generate Figs. 1b and 1e are shown in Supplementary Fig. 5.

### Flow cytometry analysis

To assess surface expression of Spike in adenoviral transduced cells, 1.25 × 10^6^ Hs 729 T cells were plated in p100 and cultured overnight. After 24 h, cells were transduced with the different adenoviral constructs at MOI 100 for 48 h. Then, cells were washed with PBS and detached with StemPro Accutase (A11105-01, Life Technologies, Carlsbad, CA, USA) and stained with Fixable Viability Stain 510 (564406; 1:1000 dilution; BD Horizon, Franklin lakes, NJ, USA) for 5 min at 37 °C. Cell were centrifuged and resuspended in PBS-BSA (0,05%) and incubated with monoclonal antibodies against anti-S1(NTD) (E7M5X; 1:200 dilution; Cell Signaling Technology, Danvers, MA, USA) or RBD region (40592-MM117; 1:250 dilution or 40592-R0004; 1:100 dilution; Sino Biological, Beijing, R.P. China) for 30 min. Then, cells were centrifuged and washed with PBS-BSA (0.05%) twice and incubated with Cy-5 Donkey anti-mouse (715-175-150; 1:400 Jackson Immunoresearch, West Grove, PA, USA) or anti-rabbit (711-175-152; 1:400 dilution Jackson Immunoresearch) secondary antibodies for 30 min. Samples were then washed twice, resuspended in PBS-BSA (0.05%) and analyzed on a FACS Aria flow cytometer (Becton-Dickinson). The data were analyzed with FlowJo software (Becton-Dickinson) using gating strategy to evaluate only intact and viable cells (Supplementary Fig. 1a).

### Mice immunization

Six- to 8-week-old SPF male BALB/c mice were obtained from the animal facility of the Veterinary School, University of La Plata, Argentina and immunized with 10^8^ or 10^9^ viral particles (vp) of Ad.C (empty vector) or each one of the vaccines in 30 μL PBS *via* i.m. injection in the hind leg. Final serum samples were obtained via the cardiac puncture of anesthetized mice. The collected whole blood was allowed to clot at 37 °C for 1 h before spinning down at 500 xg for 10 min. The clarified sera were stored at −20 °C. For booster shots, BALB/c mice were immunized with 10^9^ viral particles (vp) of CoroVaxG.3. Twenty-eight days later mice were immunized with 10^9^ viral particles (vp) of either CoroVaxG.3, CoroVaxG.3-D.FR or CoroVaxG.3-O.FR. Animals were bled 14 days after the booster dose. Animal studies were carried out following the recommendations of the Guide for the Care and Use of Laboratory Animals of the National Institutes of Health. The protocol was approved by the Institutional Committee for Care and Use of laboratory Animals of the Leloir Institute (CICUAL protocol ID 97).

### ELISA

Animal sera were evaluated for SARS-CoV-2-S-specific IgG antibodies using ELISA. Briefly, ELISA plates (BRANDplates®, immunoGrade, BRAND GMBH + CO KG) were coated with 100 ng of the recombinant ectodomain of SARS-CoV-2 Wu-1 (Sino Biological 40589-V08B1), Delta (Sino Biological 40589-V08B16) or BA.1 Spike protein (Sino Biological 40589-V08H26) per well overnight at 4 °C in 50 μL PBS and then blocked with PBS-T/3% BSA (blocking buffer) for 1 h. Following the procedure as described, bound-specific IgG was detected with an HRP-conjugated goat anti-mouse IgG H&L antibody (ab6789, Abcam) diluted at 1: 10,000 in a blocking buffer. Wu-1-S-specific IgG1e3 mAb (Invivogen, Waltham, MA, USA) and SARS-CoV-2 Spike Antibody, Omicron Reactive, Mouse Mab 40592-MM117 (Sino Biological) were used as positive controls. Color development was performed by the addition of 50 μL of TMB Single Solution (Thermo Fisher Scientific, Carlsbad, CA, USA). After 8 min, the enzyme reaction was stopped with 50 μL of 1 M sulfuric acid per well, and the absorbance was measured in a Bio-Rad Model 550 microplate reader (Bio-Rad Laboratories, Hercules, CA, USA). The sera were assayed in duplicates, and the antibody titer represents the last reciprocal serum dilution above blank.

### IFN-γ ELISPOT

Spleens were removed from vaccinated or control BALB/c mice and splenocytes were isolated by disaggregation through a metallic mesh. After RBC lysis (Biolegend, San Diego, CA, USA), resuspension and counting, the cells were ready for analysis. The IFN-γ secreting cells were assessed using the ELISPOT mouse IFN-γ kit (R&D Systems) according to the manufacturer’s protocol. The cells were cultured for 18 h at 5 ×10^5^ cells per well with 2 μg/mL of a peptide pool consisting mainly of 15-mers (overlapping by 11 amino acids) covering the immunodominant sequence domains of the SARS-CoV-2 Spike protein (PepTivator® SARS-CoV-2 Prot_S; Miltenyi Biotec, Bergisch, Gladbach, Germany). The number of spots was determined using an automatic ELISPOT reader and image analysis software (CTL-ImmunoSpot® S6 Micro Analyzer, Cellular Technology Limited (CTL), Cleveland, OH, USA).

### In vivo challenge studies with VOC

Hemizygous B6.Cg-Tg(K18-ACE2)2Prlmn/J (K18-hACE2) (JAX stock #034860) were obtained from The Jackson Laboratory (Bar Harbor, Maine, US)^62–64^. Animals were bred and housed at the Center for Comparative Medicine, National University of the Litoral. Six- to eight-month-old K18-hACE2 mice were immunized with 10^9^ viral particles (vp) of each vaccine in 30 μl PBS via an i.m. injection in the hind leg. For virus challenge, mice were anesthetized (ketamine/xylazine) and infected intranasally with 50 μl containing 1×10^4^ TCID_50_ of the Gamma VOC, 5×10^5^ TCID_50_ of the Delta VOC and 3.5×10^4^ TCID_50_ of Omicron BA.1. The viral dose was selected for each single VOC from preliminary studies in aged K18-hACE2 mice immunized with different doses of viable SARS-CoV-2 variants. Gamma and Delta VOC had a similar impact on mice health, while Omicron BA.1 infection caused attenuated disease^34,56^.

Clinical signs of disease (weight loss, rapid breathing, hunched posture and inactivity) were monitored daily until day 14 postinfection or before, if mice reached the endpoint criteria. Lung and brain were harvested at day 4 for viral titer (left half) and histopathological analyses (right half). To obtain nasal airway lavage samples a 24 G IV catheter was inserted into the trachea towards the pharyngeal region and the upper respiratory tract was rinsed with 300 µl of PBS. The fluid coming from the nostrils was collected and stored at −80 °C until use. For the virus challenge studies, K18-hACE2 mice were delivered from the animal facility of the Center for Comparative Medicine to the Biological Containment Operational Unit of Malbrán Institute, BSL3A animal facility. All procedures were performed in strict accordance with the recommendations of the Guide for the Care and Use of Laboratory Animals of the National Health Institute. All protocols were approved by the Animal Experimentation Ethics Committee of the Leloir Institute (Protocol ID #97. July 2021, modification Nov 2021) and were carried out in accordance with the ARRIVE guidelines and the SOPs of the Malbrán Institute. Every effort was made to minimize animal suffering.

### Viral load assessment

Viral titers were determined in lung and brain samples. Tissues were weighed and stored at −80 °C in DMEM. Homogenized tissue was centrifuged 10 min at 500 xg at 4 °C and the supernatant was collected. RNA was extracted the QIAamp Viral RNA Mini Kit (Qiagen). SARS-CoV-2 N and E gene copies were determined by qRT-PCR using LightMix® Modular SARS-CoV (COVID19) kit (TIB MOLBIO) according to the manufacturer’s protocol. Results are presented as the log_10_ of the number of copies per mg of tissue sample.

### Histopathological studies

Tissues were collected at necropsy and lung, brain and duodenum samples were fixed in 4% buffered formaldehyde for 8–10 h at room temperature and then washed in PBS. Later, fixed tissues were dehydrated in an ascending series of ethanol, cleared in xylene and embedded in paraffin. Sections (4 μm thick), obtained by rotative microtome, were mounted on slides treated previously with 2% (v/v) 3-aminopropyltriethoxysilane in acetone (Sigma-Aldrich, Saint Louis, MO, USA) and initially stained with hematoxylin-eosin for the histopathology analysis.

Deparaffinized slides were used for immunohistochemical staining. Briefly, CD3, but not the SARS-CoV-2 N protein, was retrieved by microwaving in 10 mM sodium citrate buffer (pH 6.0). Endogenous peroxidase activity was inhibited with 3% (v/v) H_2_O_2_ in methanol, and nonspecific binding blocked with 10% (v/v) normal goat serum in PBS. The primary antibodies against CD3 (polyclonal rabbit A0452, DAKO) and against the N SARS-CoV-2 protein (polyclonal rabbit 40143-T62, Sino Biological) were diluted 1:400 in PBS-BSA 1% - Tween 0.5%. The antibodies were incubated for 18 h at 4 °C and then for 30 min at room temperature with biotinylated secondary antibodies. The antigens were visualized using the CytoScan HRP Detection System with 3,3′-diaminobenzidine (DAB; Liquid DAB-Plus Substrate Kit; Invitrogen) as the chromogen. As a control, adjacent sections were subjected to the same procedure, replacing primary antibodies with rabbit nonimmune sera. Sections were examined by a qualified veterinary pathologist who was blinded to the animal and treatment groups.

A score of 0 to 3 based on absent, mild, moderate, or severe degree was used to describe and semiquantify pulmonary pathology injuries such as: overall lesion extent, type II pneumocyte, alveolar/bronchiolar epithelium and BALT (bronchus-associated lymphoid tissue) hyperplasia; congestion, hemorrhages and interstitial pneumonia. A cumulative pathology score was obtained for each animal. A similar scoring system was applied to evaluate lesions in the brain and the duodenum, and for the positive immunohistochemical staining of the N SARS-COV-2 protein and CD3 expression.

### Construction of VOC-matched pseudoviruses and neutralization assays

The full-length cDNA of the different Spike was constructed as follows: to introduce each Spike variant into the pcDNA-3.1, the pcDNA-3.1-D614G-Spike^28^ was restricted with BamHI/EcoRV or BamHI/SwaI, to delete the section of the Spike sequence to be replaced. Simultaneously, the Spike region was amplified in several overlapping fragments using primers containing the amino-acid changes corresponding to each VOC. Finally, the vector fragment and the Spike fragments were reassembled using Gibson assembly, and confirmed by BS-sequencing (CELEMICS, Seul, Korea). The pseudoviral particles (PVs) containing the different SARS-CoV-2 protein variants were generated as described^28^. Basically, we generated a replication-defective Vesicular Stomatitis Virus (VSV) PV in which the backbone was provided by a pseudotyped DG-luciferase (G*DG-luciferase) rVSV (Kerafast, Boston, MA, USA), that packages the expression cassette for firefly luciferase instead of VSV-G in the VSV genome. HEK293T cells growing in Optimem media (Gibco, Whaltman, MD, USA) with 2% of FBS were transduced with G*DG-VSV at a multiplicity of infection of 4. Twenty minutes later, the cells were transfected with 30 μg of pcDNA-3.1-Spike-D614G, using Lipofectamine 3000 (Thermo Fisher Scientific, Carlsbad, CA, USA) and incubated for 6 h at 37 °C, 5% CO_2_. Then, the cells were washed 4 times with PBS in order to remove all the residual G*DG-VSV, and cultured in complete media at 37 °C, 5% CO_2_. After 48 h the supernatant containing the PVs was collected, filtered (0.45-μm pore size, Millipore) and stored in single-use aliquots at −80 °C. The 50% tissue culture infectious dose (TCID_50_) of SARS-CoV-2 PV was determined in sextuplicates and calculated using the Reed–Muench method.

The neutralization assays were performed as previously described^28^. Briefly, 50 µL of serially diluted mouse sera were combined with 65 TCID_50_ PVs in 50 µL of complete medium (DMEM supplemented with 10% FBS and non-essential aminoacids) in 96 well plates (Greiner Bio-One, Germany) and incubated at 37 °C, 5% CO_2_ for 1 h. Next, 100 µL of 5 × 10^5^/mL HEK293T-ACE2 cells were added to the pseudovirus–serum mixture and incubated at 37 °C, 5% CO_2_ for 20–24 h. The conditions were tested in duplicate wells on each plate, and a virus control (VC = no sera) and cell control (CC = no PV) were included on each plate in 6 wells each to determine the value for 0 and 100% neutralization, respectively. The media were then aspirated from the cells, and the Firefly luciferase activity was determined with the Luciferase Assay System (Promega) as recommended by the manufacturer. The percentage of inhibition of infection for each dilution of the sample is calculated according to the RLU values as follows: % inhibition = [1 − (average RLU of sample − average RLU of CC)/(average RLU of VC − average RLU of CC)] × 100%. On the basis of these results, the ID_50_ of each sample was calculated by the Reed–Muench method^65^.

### Antigenic cartography

Antigenic maps were constructed as previously described^66,67^ with the online tool Acmacs (https://acmacs-web.antigenic-cartography.org). Basically, this algorithm uses multidimensional scaling to locate antigens and sera in a map to represent their antigenic relationships. The maps were generated with ID_50_ titers obtained from vaccinated BALB/c mice. One antigenic unit in the map (the space between two grid lines) corresponds to a two-fold dilution in the neutralization assay. Variants were considered to belong to different antigenic clusters when antigenic distance was at least 3 units (i.e., an 8-fold change in neutralization titer), based on the criteria used for Influenza viruses^68^.

### Statistical analysis

For S-specific binding antibodies as measured by ELISA and ID_50_s as measured by PBNA, statistical differences between immunization regimens were determined by a Kruskal-Wallis test with Dunn’s multiple comparisons *a posteriori*. Statistical analysis was performed using ANOVA test with Bonferroni’s multiple comparisons *a posteriori* when data follow a normal distribution. Brown-Forsythe and Welch correction was applied to the parametric ANOVA test when values where not homoscedastic. Kruskal-Wallis test with Dunn´s multiple comparisons *a posteriori* was used to analyze the data didn´t follow normality. Statistical differences for body weight change curves were performed using Two-way ANOVA with Bonferroni’s test a posteriori to compare vaccinated with unvaccinated mice for every time point. All analyses were conducted using GraphPad Prism software (version 8.2). Statistical significance was accepted when *p* < 0.05. The statistical tests used are indicated in each figure legend.

### Reporting summary

Further information on research design is available in the Nature Research Reporting Summary linked to this article.

### Supplementary information

Supplemental Material

Reporting Summary

## Data Availability

Authors can confirm that all relevant data are included in the paper and/or its supplementary information files. All the materials are available through an MTA agreement.
