# Supplementary material for: Cross-protection and cross-neutralization capacity of ancestral and VOC-matched SARS-CoV-2 adenoviral vector-based vaccines
Source: NPJ Vaccines. 2023 Oct 4;8:149. doi: 10.1038/s41541-023-00737-4 (PMC10550992; doi:10.1038/s41541-023-00737-4)
Supplement: Supplementary file 1 — Supplemental Material [file 41541_2023_737_MOESM1_ESM.pdf]

Supplementary Information

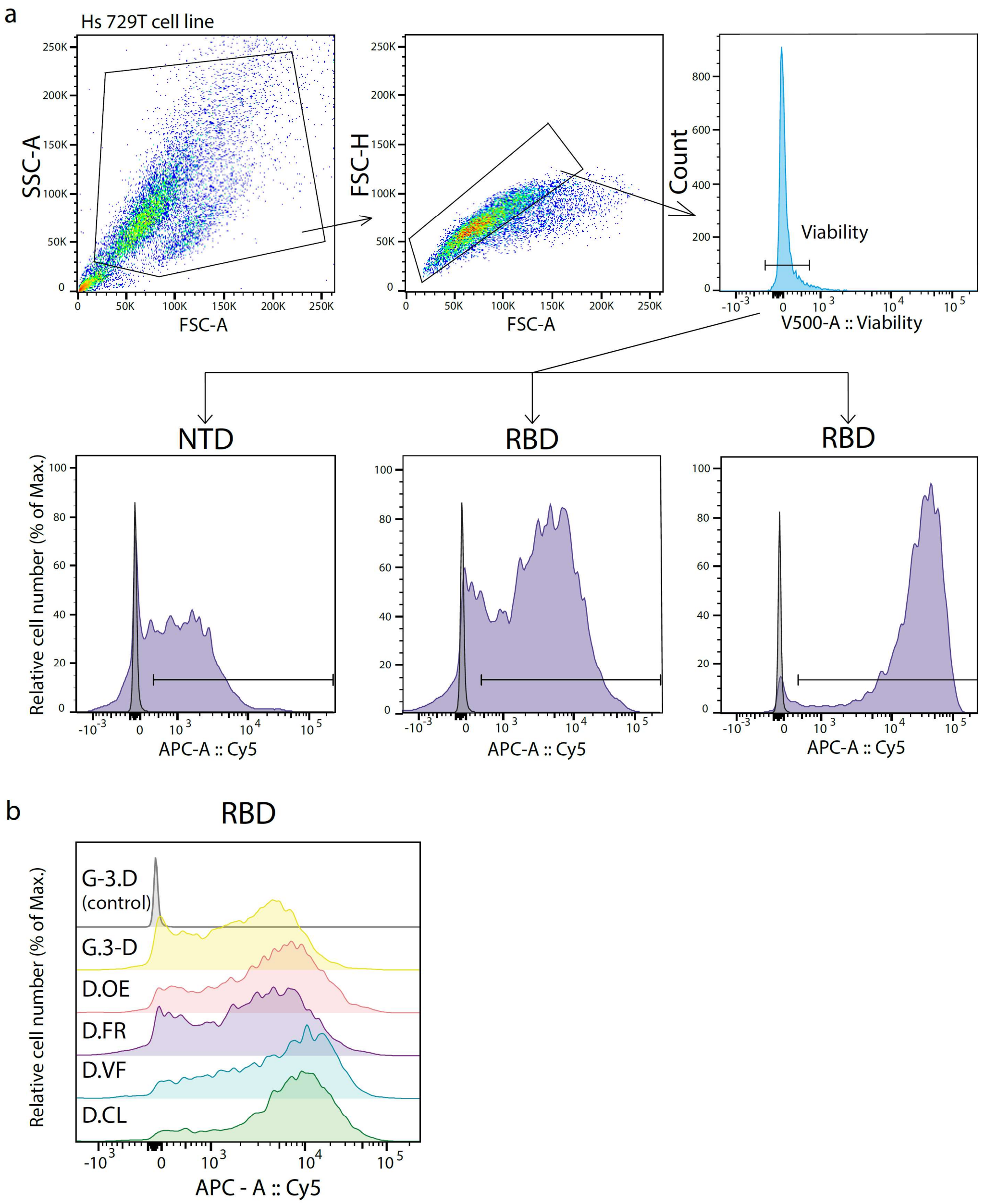

Supplementary Fig. 1. Cell surface expression of Spike following *in vitro* transduction with each vaccine candidate. Hs 729T cells were transduced with each of the vaccines at MOI 100; 48 h later Spike expression was detected by flow cytometry using the gating strategy described in the Methods section (a). (b) Results obtained using an anti-RBD monoclonal antibody (Sino Biological, 40592-MM17).

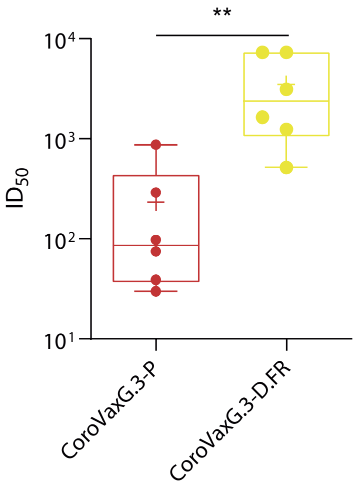

Supplementary Fig. 2. Vaccines' protection from a challenge with the Delta VOC. Transgenic mice vaccinated at day -28 were challenged with Delta at day 0. Serum samples were taken before challenge and assessed for neutralization titer against Delta PsVs. Differences between experimental groups were analyzed by Mann-Whitney test; ** $p < 0.01$. The box plots show the median, 25th and 75th percentiles, and the whiskers show the range.

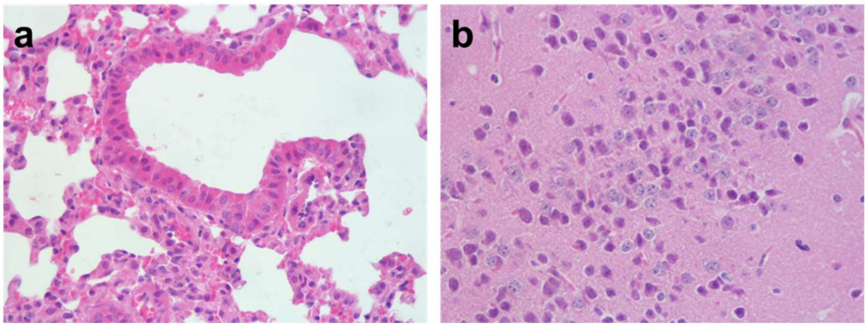

Supplementary Fig. 3. Immunopathological analysis of vaccinated unchallenged mice. Transgenic mice were vaccinated with the different vaccine candidates and stained with hematoxylin-eosin for the histopathology analysis. The panels show a representative example of the lung (a) and brain (b) of mice vaccinated with CoroVaxG.3-D.FR, which confirm the absence of tissue injury in either organ.

Figure S3

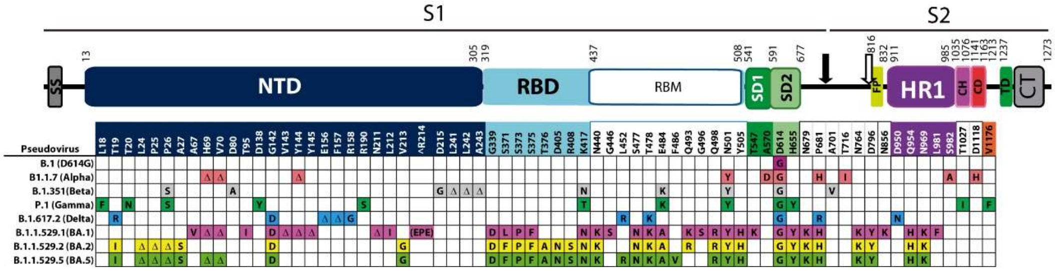

Supplementary Fig. 4. Schematic representation of Spike sequences of SARS-CoV-2 VOC-based pseudoviruses used for Pseudovirus-based Neutralization assays. For further details, see the legend to Figure 1a.

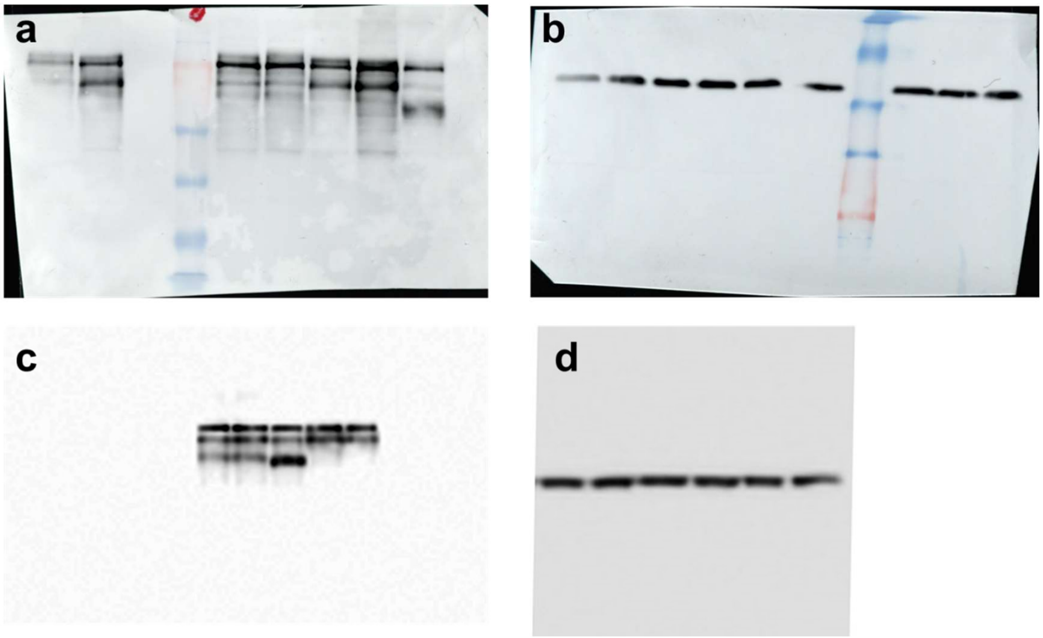

Supplementary figure 5. Uncropped and unprocessed images used to generate Fig. 1b and 1e, showing viral Spike expression in Hs 729T cells. Panels correspond to: Fig. 1b anti-Spike blot (a); Fig. 1b anti-tubulin blot (b); Fig. 1e anti-Spike blot (c); Fig. 1e anti-tubulin blot (d).

Supplementary Table 1. Geometric Mean Neutralization Titers of the variant-matched vaccines against SARS-CoV-2 VOC-based pseudoviruses.

		ID$_{50}$ GMT against VOC (95% CI)							
	Vaccine	D614G	Alpha	Beta	Gamma	Delta	BA.1	BA.2	BA.5
	CoroVaxG.3	**1085** (383-3072)	**744.6** (206.4-2686)	**392** (127.7-1203)	**552.4** (171.2-1783)	**138.8** (99.1-194.2)	**33.41** (28.84-38.71)	**33.99** (24.03-48.07)	**32.96** (25.39-42.78)
	CoroVaxG.3-P	**752.2** (287.5-1968)	**519** (137.8-1955)	**604.9** (254.1-1440)	**2243** (473.4-10630)	**204.1** (81.05-514.1)	**104.3** (21.67-502.1)	**32.82** (25.58-42.11)	**30** (30-30)
	CoroVaxG.3-D	**525.8** (109-2537)	**506.1** (148-1731)	**444.8** (80.59-2455)	**504.5** (191.9-1327)	**1238** (644.4-2377)	**96.43** (16.02-580.6)	**33.59** (27.19-41.5)	**38.54** (19.22-77.28)
	CoroVaxG.3-D.FR	**2249** (571.9-8843)	**705.6** (415.4-1198)	**506.7** (195.8-1311)	**786.4** (277.2-2231)	**5277** (3170-8782)	**84.01** (40.23-175.4)	**62.92** (19.45-203.5)	**42.01** (20.23-87.23)
	CoroVaxG.3-O.FR	**33.49** (24.67-45.45)	**30** (30-30)	**34.58** (23.31-51.28)	**35.78** (25.98-49.29)	**39.83** (23.37-67.88)	**1438** (654.1-3159)	**116.3** (59.68-226.7)	**48.39** (26.5-88.36)
Prime + boost	CoroVaxG.3 + CoroVaxG.3	**5311** (2670-10564)	**3504** (1255-9785)	**5588** (1671-18684)	**5834** (1433-23746)	**897.6** (349.5-2305)	**261.6** (72.99-937.4)	**63.22** (32.21-124.1)	**33.23** (25.02-44.12)
Prime + boost	CoroVaxG.3 + CoroVaxG.3-D.FR	**14869** (4379-50485)	**18669** (6996-49821)	**20120** (11374-35592)	**20447** (6408-65241)	**23333** (3291-165424)	**852.2** (224.7-3233)	**225.1** (20.71-2446)	**130.1** (16.6-1020)
Prime + boost	CoroVaxG.3 + CoroVaxG.3-O.FR	**4326** (1076-17388)	**6903** (1136-41966)	**2876** (560.1-14769)	**3816** (573-25410)	**5823** (2445-13870)	**9662** (2651-35223)	**305.8** (55.36-1689)	**141** (24.45-812.6)
